# One size does not fit all: An *in vitro* evaluation of the effects of bezafibrate and medroxyprogesterone acetate on human SH‐SY5Y and U‐87 MG cancer cells

**DOI:** 10.1002/2211-5463.70259

**Published:** 2026-04-21

**Authors:** Abhishek Kharawatkar, Francesco Michelangeli, Farhat Latif Khanim, Christopher Martin Bunce, William Eustace Basil Johnson

**Affiliations:** ^1^ Chester Medical School University of Chester UK; ^2^ Biomedical Sciences University of Birmingham UK; ^3^ Biosciences, University of Birmingham UK

**Keywords:** cancer cell growth, drug repurposing, reactive oxygen species, SH‐SY5Y neuroblastoma, Stearoyl‐CoA‐desaturase 1, U87‐MG glioblastoma

## Abstract

Here, we have examined the effects of the repurposed drugs bezafibrate (BEZ) and medroxyprogesterone acetate (MPA) singly and in combination (BaP) on a neuroblastoma (SH‐SY5Y) and a glioblastoma (U‐87 MG) cell line. BaP was previously shown to inhibit the growth of blood and bone cancers through the generation of reactive oxygen species (ROS) and by targeting lipogenesis. Similarly, in our study, BaP inhibited cell proliferation and induced cell death in both neuroblastoma and glioblastoma cells more effectively than single BEZ or MPA drug treatments, albeit less effectively than in blood cancers. Furthermore, we observed significant increases in ROS levels in both cancer cell lines and reductions in the levels of the lipogenic enzyme, stearoyl‐CoA‐desaturase 1 (SCD1). Supplementation with the SCD1 product, oleic acid (OA), moderately abrogated the inhibitory effects of BaP on neuroblastoma proliferation. However, this effect was not seen in glioblastoma cultures, where OA supplementation of BaP‐treated cells was associated with further decreases in cell proliferation. Lastly, we show that a clinically achievable BaP concentration enhanced the antiproliferative effects of temozolomide on glioblastoma cells. These findings show that drugs that have been successfully repurposed to potentially treat some types of cancers may have use in other cancers, but that their efficacy and mechanisms of action do not necessarily translate from one cancer type to another. Thus, successful drug repurposing requires investigation and optimisation on a case‐by‐case basis.

## Graphical abstract

Drugs previously repurposed to target blood cancers reduced neuroblastoma and glioblastoma cell growth and viability. However, their levels of anticancer activity were different and their clinical application may be problematic due to side effects at effective doses. These results demonstrate the need to investigate and further optimise the anticancer effects of repurposed drugs on a case‐by‐case basis.

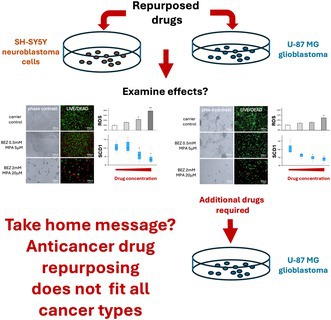

**Abbreviations**
AML: acute myeloid leukaemia
BaP: combined bezafibrate and medroxyprogesterone acetate
BEZ: bezafibrate
BL: Burkitt's lymphoma
CNS: central nervous system
DMEM/F12: Dulbecco's Modified Eagles Medium (DMEM)/F‐12
DMSO: dimethylsulphoxide
ECL: enhanced chemiluminescence
FBS: foetal bovine serum
GSC: glioblastoma stem cell
ITS: insulin, transferrin, selenium
MPA: medroxyprogesterone acetate
NEAA: nonessential amino acids
OA: oleic acid
PARP1: poly (ADP‐ribose) polymerase 1
PBS: phosphate‐buffered saline
Pen/strep: penicillin/streptomycin
ROS: reactive oxygen species
SCD1: stearoyl‐CoA‐desaturase 1
SD: standard deviation
SEM: standard error of the mean
TBaP: combined temozolomide, bezafibrate and medroxyprogesterone acetate
TMZ: temozolomide
UFA: unsaturated fatty acid
V‐BaP: combined valproic acid, bezafibrate and medroxyprogesterone acetate

Drug repurposing has gained attention in recent years due to the reduced costs and known safety profiles of previously established drugs, and the possibility that well‐researched and common mechanisms of drug action apply across different indicated diseases [1, 2]. For example, the combination of the lipid lowering drug, bezafibrate (BEZ), and the contraceptive drug, medroxypregesterone (MPA), combined as BaP, showed preclinical promise against acute myeloid leukaemia (AML) [3], chronic lymphocytic leukaemia [4], non‐Hodkin's lymphoma, including Burkitt's lymphoma (BL) [5], and some evidence of efficacy in the phase II trials in AML [6] and endemic BL [7]. BaP treatment induced metabolic changes in haematological cells by increasing levels of reactive oxygen species (ROS) and altering prostaglandin synthesis, and by decreased lipogenesis of unsaturated fatty acids (UFAs) as a result of lower levels of the lipogenic enzyme, stearoyl‐CoA‐desaturase 1 (SCD1) [3, 4, 8]; this was associated with reduced cell proliferation, increased cell differentiation and increased cell death. The importance of reduced SCD1‐mediated lipogenesis of UFAs was demonstrated by the fact that BaP‐treated AML and endemic BL cultures are rescued by supplementing culture medium with the SCD1 product, OA [8]. Later studies demonstrated that when BaP was combined with valproic acid, this combination (V‐BaP) enhanced the anticancerous effects of BaP on AML cells [9] and inhibited the growth of human osteosarcoma cell lines, SAOS2 and MG‐63, without marked deleterious effects on their normal mesenchymal counterparts [10], which again was associated with increased ROS levels [9] and reduced levels of the lipogenic enzyme, SCD1 [9, 10]. These studies have contributed to the growing hypothesis that a variety of cancer types are commonly associated with altered metabolic profiles, where enhanced ROS and reduced lipogenesis represents an attractive and broad target for anticancer therapies [11, 12].

Human neuroblastomas are peripheral neuroendocrine tumours, most commonly seen in children. Neuroblastoma treatments combine tumour resection, radiotherapy and chemotherapy, but patient prognosis remains poor with a less than 50% survival rate at 5 years [13, 14]. The problems of developing new treatments for neuroblastoma relate to its complexity and heterogeneity. Oncogenesis and disease progression have been linked with *MYCN* amplification and *ALK* mutations and with dysregulated PI3K/AKT, Ras/Raf, JAK‐STAT, NF‐κB and GSK3*α*/*β* signalling [14]. Immature, less differentiated tumours are more aggressive and more likely to develop drug resistance [13, 14]. However, highly aggressive neuroblastomas exhibit increased lipogenic supply of UFAs [15, 16] and inhibition of SCD1 activity through reduced expression and pharmacological intervention recently was associated with reduced neuroblastoma growth and increased cell death [16].

Glioblastomas are aggressive tumours of the CNS seen in older patients, which carry a very poor prognosis. The median lifespan of patients with glioblastoma multiforme is 18 months with a 5 year lifespan of only 5% [17]. Glioblastoma treatment includes resection, radiotherapy and chemotherapy, where the standard drug treatment is temozolomide (TMZ) [17, 18]. TMZ is an alkylating agent that targets DNA synthesis; however, TMZ resistance arises in approximately 50% of glioblastomas. TMZ resistance has been associated principally with increased DNA repair capacity, increased drug exporter levels, and an emergence of a glioblastoma stem cell (GSC) phenotype [19]. Importantly, elevated levels of SCD1 have been reported in GSCs and TMZ resistant glioblastoma [20, 21], and inhibiting SCD1 activity was shown to reduce GSC growth [21, 22], suggesting that altered lipogenesis plays an essential role in disease progression here too.

This study, therefore, has explored the application of BEZ and MPA, shown particularly to target blood cancer cells through elevated levels of ROS [3, 8] and blood and bone cancer cells through SCD1‐mediated lipogenesis [8, 9, 10] by testing the effects of these repurposed drugs on SH‐SY5Y neuroblastoma and U‐87 MG glioblastoma cells.

## Methods

### Cell culture

SH‐SY5Y neuroblastoma cells (ECACC 94030304) were cultured in Dulbecco's Modified Eagles Medium (DMEM)/F‐12 supplemented with 15% fetal bovine serum (FBS), 1% nonessential amino acids (NEAA), 1% insulin, transferrin, selenium (ITS) and 1% penicillin/streptomycin (pen/strep) (Thermo Fisher, UK). U‐87 MG glioblastoma cells (ECACC 89081402) were cultured in DMEM/F12 supplemented with 10% FBS, 1% NEAA and 1% pen/strep, passaged at 80% confluence and sub‐cultured at 5 × 10^3^ cell/cm^2^. Stock cultures were routinely passaged by trypsinisation; stock culture viability was always greater than 95%, and only cultures below passage 25 were used. These cell lines were deposited by the University of Leeds (SH‐SY5Y) and AVRI Pirbight (U‐87 MG) and have been authenticated by the Cell Culture Collection of the UK Health Security Agency (ECACC) using STR profiling. All experiments were performed with mycoplasma‐free cells.

### Drug preparations and treatments

BEZ was prepared as a 0.5 m stock solution dissolved in dimethylsulphoxide (DMSO), whilst MPA was prepared as a 5‐mm stock solution dissolved in ≥ 99.9% methanol, following supplier guidelines (Sigma‐Aldrich, UK). BEZ and/or MPA were added to standard culture media to form concentrations of 2 mm BEZ, or 20 μm MPA, or a combination of 2 mm BEZ plus 20 μm MPA (BaP), then doubling dilutions were performed to a lowest drug concentration of 0.25 mm BEZ, or 2.5 μm MPA, or combined 0.25 mm BEZ plus 2.5 μm MPA (BaP), respectively. TMZ (Sigma‐Aldrich) was prepared in DMSO at a stock concentration of 200 mm and was added to media at concentrations of 25 μm–200 μm. In some experiments, BaP‐treated SH‐SY5Y and U‐87 MG cultures were supplemented with 100 μm–300 μm OA (in phosphate‐buffered saline, PBS; Sigma‐Aldrich), as previously reported [8]. Carrier control media were prepared with dilutions of DMSO and/or methanol or PBS at dilutions equivalent to the highest concentrations of the respective drug treatments.

SH‐SY5Y and U‐87 MG cells were seeded at 5000 viable cells/well in 100 μL of standard culture media in 96‐well plates (StarLabs, UK) with at least three replicate wells for each drug concentration. Plates were incubated at 37 °C in a 5% CO_2_ humidified incubator overnight; then, the standard culture media were replaced with drug‐treated media and the plates further cultured for 5 days, with an additional 100 μL of drug‐treated media (versus carrier control media) added on Day 3. In separate experiments, U‐87 MG cells were seeded at 5000 viable cells/well in 100 μL standard culture media, left overnight, and then, after removing the standard media, treated with 100 μL of media containing TMZ alone or with a combination of 0.5 mm BEZ plus 5 μm MPA plus TMZ (TBaP), with 100 μL of fresh drug‐treated media added on Day 3. All experiments were harvested on Day 5 (unless otherwise stated).

### Cell proliferation

Viable cell numbers were determined by MTS assay using CellTiter96, Aqueous One Solution Cell Proliferation Assay Kit (Promega, UK) according to the manufacturer's protocol. Spectrophotometry for viable cells was combined with imaging under phase contrast microscopy, as reported previously [10]. Absorbance levels were normalised to those levels detected in carrier control cultures for each independent experiment performed.

### LIVE/DEAD staining

Cell viability was assessed by LIVE/DEAD staining (LIVE/DEAD Cell Double Staining Kit; Sigma‐Aldrich, UK) according to the manufacturer's instructions and as reported previously [23]. The proportions of SH‐SY5Y and U‐87 MG cells that were either viable (green fluorescence, LIVE) or nonviable (red fluorescence, DEAD) were determined by scoring cells using a 10× objective under phase contrast and fluorescence microscopy.

### SCD1 immunoblotting

Immunoblotting for SCD1 levels was performed at 1 day after BaP drug treatments (versus controls), as reported previously [8, 10]. Briefly, equal amounts of cell‐extracted proteins (30 μg) underwent SDS/PAGE on gradient gels (Bio‐Rad, UK), were transferred onto PDVF membranes, blocked in blocking buffer (5% dried milk in 0.1% Tween/PBS) and incubated with mouse monoclonal anti‐*β*‐actin antibodies (A5441; Sigma‐Aldrich). After washes (0.1% Tween/PBS washing buffer), *β*‐actin immunodetection was performed with goat anti‐mouse HRP‐conjugated antibodies (ab 205 719; Abcam Ltd., UK) and enhanced chemiluminescence (ECL) (SupraSignal™ West Pico Chemiluminescent Substrate, Thermo Fisher). *β*‐actin levels were determined using a transilluminator G:BOX Chemi–XRQ, SYNGENE and GENESYS v1. Next, membranes were incubated in a mild stripping buffer [10], with further washes, re‐blocked and re‐probed with monoclonal anti–SCD1 antibodies (ab 19 862; Abcam Ltd), followed by repeated washes, incubation with goat anti‐mouse HRP‐conjugated antibodies and ECL based immunodetection. Scanning densitometry of *β*‐actin and SCD1 immunoreactivity was performed using the imagej software, and SCD1 levels were normalised to *β*‐actin levels, where carrier control cultures were considered 100% [8, 10].

### ROS assays

ROS levels were measured at 1 day after BaP treatment using a commercial kit (DCFDA/H2DCFDA Cellular ROS Assay Kit; Abcam Ltd) according to the manufacturer's instructions. In brief, SH‐SY5Y and U‐87 MG cells were seeded in standard culture media, incubated overnight, treated with BaP for 24 h and then assayed for ROS assay as follows: media were removed and the cells were washed, DCFDA solution was added to each well and plates were incubated for 45 min; DCFDA staining solution was removed, cells were washed then the extent of fluorescence was measured using a fluorescence reader with 520 nm emission and 485 nm excitation filters. ROS levels were normalised to the numbers of viable cells present (determined at the same 24 h time point by MTS assays) and as a % of carrier control levels.

### Statistical analysis

At least three independent experiments were performed with at least three internal repeats for assays of cell proliferation, cell viability and ROS generation. Statistical analysis was performed using the spss software (version 28.0) and Jamovi (version 2.3.28). Data were tested for normal distributions, then analysed using nonparametric Kruskal–Wallis ANOVAs and post hoc Mann–Whitney *U*‐tests. Data have been shown as means ± standard error of the mean (SEM) except for SCD1 protein levels, which are shown as means ± standard deviations (SD) (in the main text) and box and whisker plots (Fig. 2). *P* values ≤ 0.05 were considered significant.

## Results

### Combined BEZ and MPA treatments were most effective at reducing SH‐SY5Y and U‐87 MG cell proliferation and culture viability

**Fig. 1:**
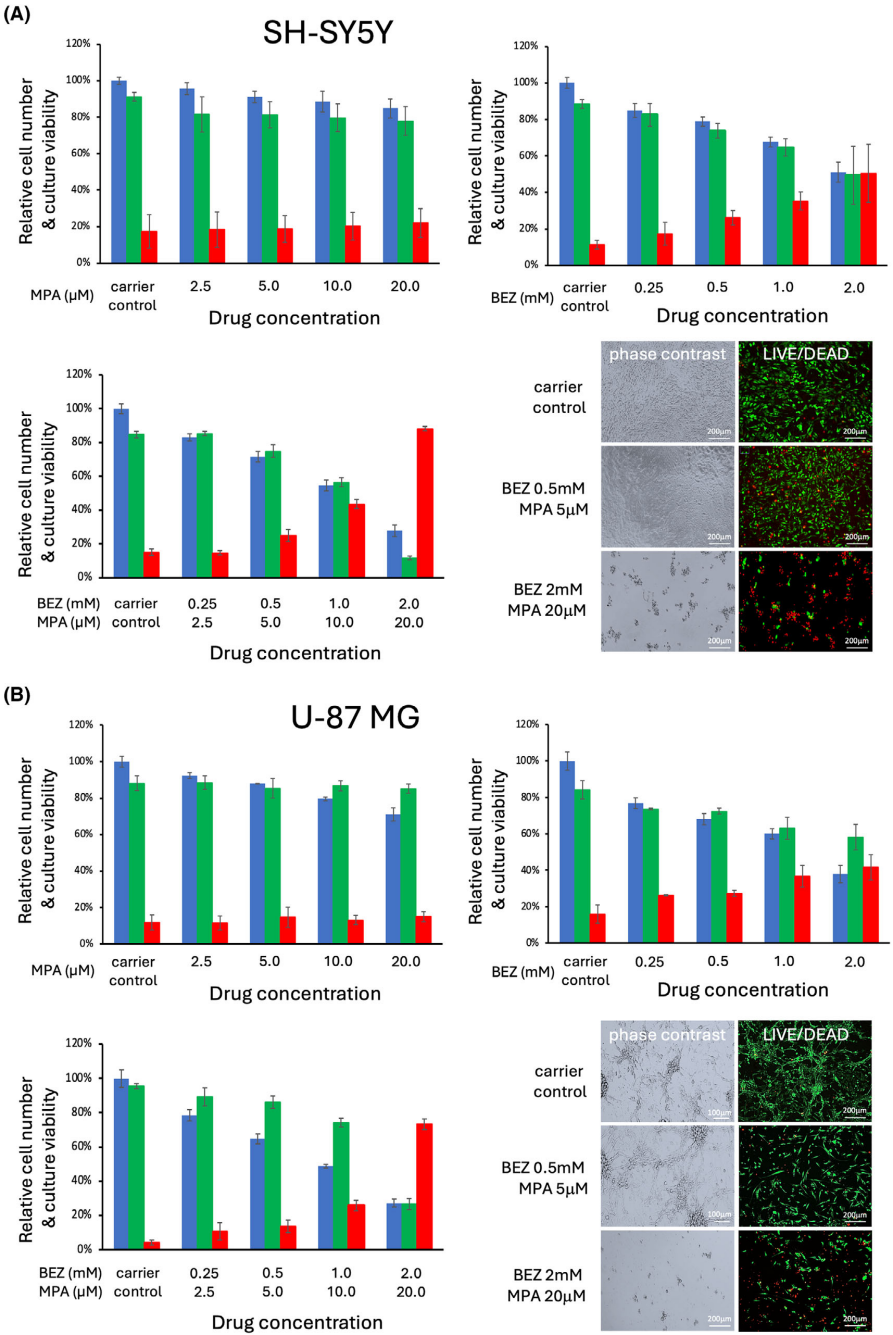
Anticancer effects of combined bezafibrate and medroxyprogesterone acetate (BaP) treatment on SH‐SY5Y and U‐87 MG cells were more effective than bezafibrate (BEZ) or medroxyprogesterone acetate (MPA) treatment alone. (A) SH‐SY5Y neuroblastoma and (B) U‐87 MG glioblastoma cell numbers relative to controls (blue bars) and % viability (green bars = viable cells, red bars = dead cells) in cultures treated with BEZ, MPA and BaP for 5 days. Inset (bottom right): representative phase contrast and LIVE/DEAD staining images in control versus BaP‐treated cultures. Data shown as means ± SEM. *N* = 3 independent experiments. Scale bars are 200 μm.

Treatments of SH‐SY5Y and U‐87 MG cells with BEZ, MPA and combined BEZ and MPA (BaP) were associated with decreased cell proliferation and decreased culture viability. The effects of BEZ alone were markedly greater than MPA alone; however, BaP treatment was most effective in reducing cell proliferation and culture viability for both cell lines (Fig. 1). At BaP concentrations of 0.5 mm BEZ plus 5 μm MPA, SH‐SY5Y and U‐87 MG cell proliferation were 72% ± 3% and 65% ± 3% of control levels, respectively. At BaP concentrations of 2 mm BEZ plus 20 μm MPA, SH‐SY5Y and U‐87 MG cell proliferation were 28% ± 3% and 27% ± 2% of control levels, respectively. Furthermore, at BaP concentrations of 0.5 mm BEZ plus 5 μm MPA, SH‐SY5Y and U‐87 MG culture viabilities were 75% ± 4% and 86% ± 4% viable, respectively, whilst at BaP concentrations of 2 mm BEZ plus 20 μm MPA, SH‐SY5Y and U‐87 MG culture viabilities were 12% ± 1% and 27% ± 3% viable, respectively. These differences in cell proliferation and culture viability in BaP‐treated cultures versus control cultures were marked and significant (nonparametric ANOVAs and Mann–Whitney *U*‐tests).

### Combined BEZ and MPA treatment was associated with increased ROS generation and decreased SCD1 levels prior to changes in cell proliferation or culture viability

**Fig. 2:**
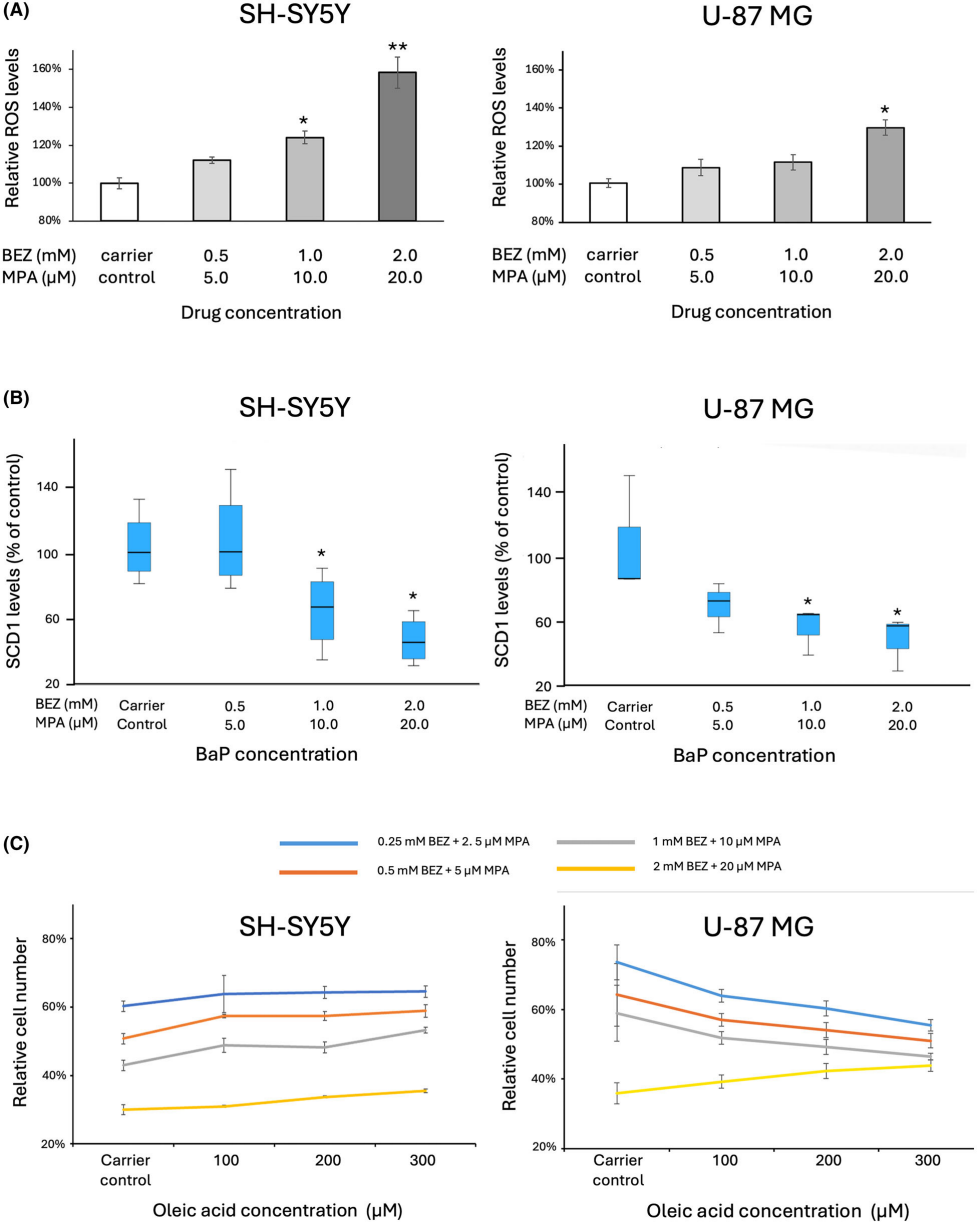
Combined bezafibrate and medroxyprogesterone acetate (BaP) treatment of SH‐SY5Y and U‐87 MG cells was associated with increased reactive oxygen species (ROS) and decreased SCD1 levels, but oleic acid (OA) supplementation did not markedly affect cancer cell growth. (A) ROS levels in cultures treated with BaP for 24 h (data normalised to control levels, shown as means ± SEM). (B) SCD1 levels in cultures treated with BP for 24 h. Data normalised to control levels, shown as box and whisker plots. (C) SH‐SY5Y and U‐87 MG cell numbers relative to controls at Day 5 of BaP‐treated cultures with OA supplementation. Data shown as means ± SEM. *N* = 3 independent experiments. *P ≤ 0.05, **P ≤ 0.01 from control values using nonparametric Kruskal–Wallis ANOVAs and post hoc Mann–Whitney *U*‐tests.

BaP treatment was associated with concentration‐dependent increases in levels of ROS in SH‐SY5Y and U‐87 MG cells at 24 h post‐treatment (Fig. 2A). At this time, in 0.5 mm BEZ plus 5 μm MPA treated cultures, ROS levels in SH‐SY5Y and U‐87 MG cells were 112% ± 2% and 112% ± 4% of control levels, respectively; whilst in BaP concentrations of 2 mm BEZ plus 20 μm MPA, ROS levels in SH‐SY5Y and U‐87 MG cells were 158% ± 8% and 130% ± 4% of control levels, respectively.

At 24 h post‐BaP treatment, there was a BaP concentration‐dependent decrease in SCD1 protein levels (Fig. 2B). At this time, SCD1 levels were 47% ± 13% and 52% ± 14% of control levels in 2 mm BEZ plus 20 μm MPA BaP‐treated SH‐SY5Y and U‐87 MG cells, respectively. There were no significant differences in SH‐SY5Y or U‐87 MG cell numbers or culture viabilities in any cultures at 24 h post‐treatment (data not shown).

### Supplementation of BaP‐treated SH‐SY5Y and U‐87 MG cultures with OA had differential effects on cell proliferation

Supplementing SY‐SY5Y cultures with increasing concentration of the monounsaturated fatty acid, OA, was associated with a moderate abrogation of the antiproliferative effects of BaP treatments. For example, in this series of experiments the highest concentrations of BaP treatment (2 mm BEZ plus 20 μm MPA) was associated with a reduction in SH‐SY5Y cell proliferation to 31% ± 0% of control levels versus 36% ± 1% of control levels when supplemented with 300 μm of OA. In contrast, supplementing BaP‐treated U‐87 MG cultures with OA was associated with further decreased U‐87 MG cell proliferation in all except the highest concentration of BaP treatment. In this series of experiments, the lowest concentrations of BaP treatment (0.25 mm BEZ plus 2.5 μm MPA) was associated with a reduction in U‐87 MG cell proliferation to 74% ± 5% of control levels versus 55% ± 2% of control levels when supplemented with 300 μm of OA (p ≤ 0.05, Mann–Whitney *U‐*test), whilst the highest concentrations of BaP treatment (2 mm BEZ plus 20 μm MPA) was associated with a reduction in U‐87 MG cell proliferation to 36% ± 3% of control levels versus 44% ± 2% of control levels when supplemented with 300 μm of OA (*P* ≤ 0.05, Mann–Whitney *U‐*test) (Fig. 2C).

### Combining TMZ with BaP was more effective than TMZ alone at reducing U‐87 MG cell proliferation, but did not decrease culture viability

**Fig. 3:**
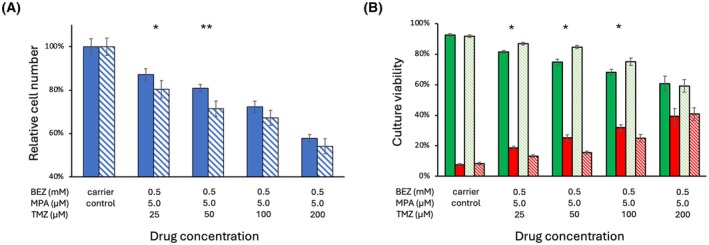
Combined bezafibrate and medroxyprogesterone acetate (BaP) combined with temozolomide (TMZ) (TBaP) was moderately more effective than TMZ alone at inhibiting U‐87 MG cell proliferation. (A) U‐87 MG glioblastoma cell numbers relative to controls in TMZ alone versus TBaP‐treated cultures at Day 5 (solid bars = TMZ alone; diagonal bars = TBaP). (B) U‐87 MG culture viability (green bars = viable cells, red bars = dead cells) in cultures treated with TMZ alone (solid bars) versus TBaP (diagonal bars). Data shown as means ± SEM. *N* = 3 independent experiments; *P ≤ 0.05, **P ≤ 0.01 using nonparametric Kruskal–Wallis ANOVAs and post hoc Mann–Whitney *U*‐tests.

TMZ treatment decreased U‐87 MG cell proliferation and culture viability in a TMZ concentration‐dependent manner. U‐87 MG cell proliferation was further reduced by the addition of 0.5 mm BEZ plus 5 μm MPA to the TMZ treated cultures. For example, treatment with 50 μm TMZ alone showed decreased U‐87 MG cell proliferation to 81% ± 2% of control levels, whereas U‐87 MG cell proliferation decreased to 71% ± 3% of control levels in cultures treated with 50 μm TMZ and 0.5 mm BEZ plus 5 μm MPA. In contrast, U‐87 MG cell viability was slightly increased when BaP was added to some of the TMZ treatments; for example, culture viability was 75% ± 2% when treated with 50 μm TMZ alone compared with a culture viability of 85% ± 1% when treated with 50 μm TMZ and 0.5 mm BEZ plus 5 μm MPA (Fig. 3).

## Discussion

Drug repurposing has been a fruitful exploration of how old drugs with established mechanisms of action and known toxicity profiles can be used to treat diseases other than those originally intended. Combined BEZ and MPA (BaP) was shown to markedly decrease the proliferation and reduce the viability of blood cancers by increased ROS generation, altered prostaglandin metabolism and reduced lipogenesis through reduced SCD1‐mediated generation of UFAs, which was enhanced by the addition of valproic acid to the repurposed drug combination (V‐BaP) [3, 8, 9]. V‐BaP was shown to reduce osteosarcoma cell growth, viability and migration, which also was associated with decreased levels of the SCD1 [10].

Here, we have sought to extend the use of repurposed BaP treatments to target human SH‐SY5Y neuroblastoma and U‐87 MG glioblastoma cells. BaP treatment was more effective than either BEZ or MPA alone in reducing the growth and viability of these neuronal cancer cells. BaP treatment also was associated with increased ROS generation and decreased levels of SCD1. Therefore, these results support the hypothesis that drugs that have been successfully repurposed from some forms of cancer can be used to target other forms of cancer, where their mechanisms of action are indicated.

However, there were important differences in the susceptibility of blood cancers [3, 8, 9] and osteosarcomas [10] to BaP treatment compared with the neuronal cancer cells. Notably, the concentrations of BaP needed to reduce SH‐SY5Y and U‐87 MG cell proliferation to less than 50% of control levels were greater than those seen in the blood and bone cancer cell lines [3, 8, 9, 10]. This is important because the effective BaP concentration used with blood cancers *in vitro* equates to the upper end of BaP dosages that can be tolerated in human drug trials [6, 7]. In addition, whilst elevated ROS levels and decreased SCD1 were seen commonly across the different cancer types, there was little if any rescue of SH‐SY5Y or U‐87 MG cells when supplementing BaP‐treated cultures with OA. This is in sharp contrast to our previous finding in AML cells and endemic BL, where the anticancer effects of BaP were abrogated by OA [8].

The roles that SCD1 may play in mediating cancer progression are known to be complex [24]. Recent studies have shown that SCD1 and SCD‐5, which is the prevalent isoform of the desaturase enzyme found in the brain, modulate the DNA damage response of glioblastoma cells by affecting poly (ADP‐ribose) polymerase 1 (PARP1) function [25]. PARP1 is the main driver of DNA damage responses in glioblastoma after radiotherapy and TMZ treatment and is upregulated in GSCs [25]. Bez is well known to elevate PARP levels [3]. However, SCD1 and SCD‐5 knockdown experiments in GSCs were also shown to decrease PARP1 levels, which in turn were associated with reduced homologous recombination activity, increased DNA damage and cell death [25]. Furthermore, an accumulation of saturated fatty acids and increased ROS levels were implicated in this interaction of SCD activity and PARP1 function. It is possible, therefore, that OA supplementation did not rescue U‐87 MG cells from BaP treatment because the effects of BaP on these cancer cells were independent of UFAs.

Lastly, our results demonstrated that BaP treatment had a modest but significant effect in inhibiting U‐87 MG cell proliferation when combined with TMZ (TBaP). This supports the combined use of BaP with TMZ even though there was a moderate but significantly increased U‐87 MG culture viability in some TBaP concentrations, suggesting that TBaP may also affect differentiation status.

There are limitations to the findings in this research. Notably, it would be informative to examine the role of increased ROS levels in inductions of SH‐SY5Y and U‐87 MG cell growth arrest and cell death by inhibiting ROS generation in the presence of BaP. Further, it would be worthwhile examining whether the protection of U‐87 MG cells from cell death in TBaP‐treated cultures was due to exit from the cell cycle and increased differentiation, for example by examining cell cycle profiles and differentiation markers. Further research to address these limitations is warranted.

Overall, however, our findings show that whilst drug repurposing to develop new anticancer therapies across different cancer types is an attractive and timely proposition, there is a need to examine both the extent of these effects and their mechanisms of action, which are likely complex, on a case‐by‐case basis.

## Funding

The authors have nothing to report.

## Conflicts of interest

The authors declare no conflicts of interest.

## Author contributions

AK, FM, FLK, CMB, and WEBJ conceived the project. FM and WEBJ designed the project. AK and WEBJ acquired the data. AK, FM and WEBJ analysed the data. FM and WEBJ supervised the study. AK and WEBJ wrote the draft paper. FLK, CMB and WEBJ provided materials for the project. All authors interpreted the data and edited the final paper.

## Data Availability

The data that support the findings of this study are available in Figs 1, 2, 3 of this article.
